# Trait Emotional Intelligence and Children’s Eating Practices

**DOI:** 10.3390/bs16020302

**Published:** 2026-02-20

**Authors:** Caterina Laganà, Eliana De Salvo, Francesco Preiti, Maria Cristina Gugliandolo

**Affiliations:** 1 Department of Health Science, University “Magna Graecia” of Catanzaro, 88100 Catanzaro, Italy; caterina.lagana@unicz.it; 2 Centre for Research and Psychological Intervention (Ce.R.I.P.), University of Messina, 98100 Messina, Italy; eliana.desalvo@unime.it; 3 Department of Clinical and Experimental Medicine, University of Messina, 98100 Messina, Italy; francesco.preiti@unime.it

**Keywords:** trait emotional intelligence, mindful eating, emotional over-eating, children

## Abstract

Introduction: Given the growing prevalence of eating-related health problems among children, it is essential to promote well-being and investigate the factors that may underlie these issues. Emotional intelligence has been identified in several studies as a protective factor for children’s psychosocial adjustment, yet its effects on eating habits remain largely underexplored. This cross-sectional correlational study aims to investigate the relationship between trait emotional intelligence, mindful eating, and emotional over-eating in children. Methods: In the present study, participants were 110 children aged between 8 and 12 years and their parents. Children completed the Trait Emotional Intelligence Questionnaire—Children short form (TEIQUE-CSF) and the Mindful Eating Questionnaire adapted for Children (MEQ-C). Parents completed the Emotional Over-eating subscale of Children’s Eating Behaviour Questionnaire (CEBQ). Results: A regression-based mediation model indicated that children’s trait emotional intelligence is positively related to mindful eating, which in turn is negatively related to emotional over-eating behaviors. The results further revealed that children with lower-than-average levels of emotional over-eating reported greater mindful eating than those with higher levels. Conclusions: These findings highlight trait emotional intelligence as a factor related to children’s eating behavior, suggesting that interventions aimed at enhancing emotional regulation skills and promoting mindful eating practices are particularly warranted in the context of heightened vulnerability to eating disorders among children.

## 1. Introduction

### 1.1. Trait Emotional Intelligence in Children

In the transition from childhood to preadolescence, which occurs approximately between the ages of 8 and 12, children begin to experience various changes from an emotional, cognitive and behavioral point of view (Mascia et al., 2023). In this age group, advances in metacognitive reflection and abstraction skills are responsible for children’s growing awareness and understanding of their own and others’ emotions, which lead to higher emotional self-regulation and emotional self-efficacy (Castro et al., 2016). In this perspective, in contrast to the conceptualization of emotional intelligence as an ability (Mayer et al., 2016), trait emotional intelligence (trait-EI) has been defined as a set of relatively stable emotional dispositions, subjective self-perceptions, and personality characteristics related to self-reported emotions (Petrides & Furnham, 2001). Research on trait-EI in children has found that children’s emotional life differs fundamentally from that of adolescents and adults (Mavroveli et al., 2009) and therefore should be addressed differently. Trait-EI in this developmental age is, in fact, divided into a set of skills subjectively perceived by the child and attributable to different functional dimensions (Mavroveli et al., 2008b). A first component concerns the ability to adapt, that is, the perception of being able to orient oneself and adapt effectively to new or changing situations. This dimension is accompanied by affective disposition, which reflects the frequency and intensity with which children report experiencing different emotional states. Emotional regulation is another central component and concerns the level at which children perceive themselves to be able to modulate and control their emotional reactions. The latter is closely associated with low impulsivity, or the perception of being able to manage one’s impulses and maintain self-regulated behaviors. Alongside intrapersonal dimensions, trait-EI also includes relational and motivational aspects. These facets concern the assessment that children provide regarding the quality of relationships established with their peers, self-esteem, defined as the perception of one’s personal value and abilities, and self-motivation, which reflects the degree to which children perceive themselves as committed, improvement-oriented and capable of sustaining investment in the activities they undertake (Mavroveli et al., 2008b). Thus, the construct of trait-EI allows a comprehensive grasp of the different ways in which individuals, and especially children, process emotional information and behave in situations of high emotional relevance (Sarrionandia & Mikolajczak, 2020). Although emotion regulation becomes increasingly sophisticated with development, dysregulated emotional processes can lead children and preadolescents to learn and use ineffective strategies that expose them to health and well-being risks (Pauletto et al., 2021; Özal et al., 2024). In fact, the literature suggests that trait-EI in children represents an important predictor of scholastic performance and social competence, and is associated with a reduced risk of depressive symptoms, loneliness and somatic disorders (Andrei et al., 2015; Davis et al., 2019; Frederickson et al., 2012; Jellesma et al., 2011; Mavroveli et al., 2009; Özal et al., 2024).

### 1.2. The Link Between Trait-EI and Eating Behaviors

Among various behavioral outcomes, specific difficulties in emotional regulation may increase the risk of developing dysfunctional eating behaviors, resulting in weight gain and childhood obesity (Favieri et al., 2021). In this regard, the most recent findings indicate that approximately 25% of the European pediatric population is overweight, including cases of obesity, underlining how eating behaviors and the determinants of their dysfunction represent critical factors to be investigated for the prevention of major pathologies of public health interest in developmental age (World Health Organization, Regional Office for Europe, 2025). Although, on the one hand, at the cultural level, the excessive availability of ultra-processed and sugary foods (Costa et al., 2021), and on the other, parenting models and practices (Kontochristopoulou et al., 2024; Li et al., 2025) represent the best-known causes of excess weight in childhood, recent studies have increasingly examined the role that the emotional dimension, and especially the difficulties of emotion regulation, can have in the development and maintenance of behaviors associated with eating disorders (Warne et al., 2023). The concept of emotional eating, for example, includes eating behaviors that do not respond to a physical need for hunger, but represent a way of dealing with emotionally difficult situations (Mentzelou et al., 2025). Children may develop a tendency to eat more in response to anxiety, sadness, frustration, or stress and use food to calm themselves or gain comfort, thus using it as an emotional regulator (Herle et al., 2018). Emotional eating has been found to be related to obesity/overweight, depression, anxiety, stress, specific dietary habits and mental disorders in children (Mentzelou et al., 2025).

Given these premises, it appears evident how greater emotional awareness, represented by trait-EI, can be configured as an important factor negatively associated with the development of emotional eating and dysfunctional eating behaviors. In fact, trait-EI has been found to be strongly related to eating disorders and related constructs, such as body uneasiness (Gugliandolo et al., 2020) and emotional eating (Zysberg, 2018), both in adolescent (Cuesta-Zamora et al., 2018; Riolo et al., 2025) and adult populations (Biolcati et al., 2021; Barberis et al., 2018). Despite this substantial body of evidence, to date, to our knowledge, there is a lack of scientific literature investigating the relationship between trait-EI and emotional eating among children.

### 1.3. The Role of the Mindful Eating Approach in the Eating Attitudes of Children

Recently, research (Minari et al., 2024; Taylor et al., 2015) has highlighted how mindful eating could be an important protective factor against the onset of dysfunctional eating behaviors.

The mindful eating approach consists of intentional attention to bodily sensations of hunger and satiety, and to thoughts and feelings related to food (Alberts et al., 2012). This eating awareness may be negatively associated with emotional eating, given that the ability to observe one’s eating behaviors reduces the automatic mechanism of “eating without thinking” to manage emotions (Hart et al., 2018; Peitz & Warschburger, 2022). Consequently, mindful eating appears to be related to reduced emotional eating (Güney & Göbel, 2025) and eating problems (Kocaadam-Bozkurt & Koksal, 2025).

Although the literature is vast regarding the protective role of mindful eating among adults and adolescents, few studies have investigated this construct among children (Kocaadam-Bozkurt & Koksal, 2025; Pierson et al., 2019), albeit with promising results, consistent with previous findings.

Furthermore, although several studies have highlighted the relationship between trait-EI and mindfulness (Miao et al., 2018), recognizing how the tendency to stay in the present moment, with a non-judgmental but open attitude to experience, is related to greater emotional self-efficacy, to date, no study has investigated the relationship between trait-EI and mindful eating.

### 1.4. Gender Differences in Variables Linked to Childhood Eating Outcomes

Although the variables under investigation—albeit with different aims—have been examined in several previous studies, findings regarding potential gender differences remain inconsistent (Labree et al., 2011). Indeed, contrary to some earlier research in childhood that reported higher BMI levels in girls than in boys (Freedman et al., 2005), the World Health Organization (WHO) currently indicates that, within the European context, boys are at a higher risk of developing obesity (13%) compared to girls (9%), likely as a result of increasing BMI levels among boys (WHO, 2025). This latter finding may reflect a tendency toward automatic, non-mindful eating behaviors (i.e., mindless eating), which some studies suggest are more prevalent in boys than in girls (Kocaadam-Bozkurt et al., 2022). However, evidence concerning gender differences in mindful eating during childhood remains inconclusive.

More broadly, child gender, when considered as a background variable, may exert an indirect influence on eating-related outcomes, as it is associated with differences in children’s levels of emotional intelligence. In this regard, some studies have reported higher levels of trait emotional intelligence in girls compared to boys (Mavroveli et al., 2009), whereas others have found no significant gender differences (Mavroveli et al., 2008b). In this vein, the review by Favieri et al. (2021), which highlights the association between difficulties in emotional regulation and an increased risk of unhealthy eating behaviors, underscores the need for a more in-depth examination of how gender may influence the relationships among these constructs. Therefore, further research is warranted to clarify gender differences in trait emotional intelligence, mindful eating, emotional over-eating, and BMI in boys and girls.

### 1.5. The Present Study

In light of the previous premises, considering that a diet influenced by emotional states represents one of the main causes of childhood obesity (Bozkurt et al., 2024; Mentzelou et al., 2025) while trait-EI and more mindful eating practices are associated with a better nutritional status in different age groups (Favieri et al., 2021; Kocaadam-Bozkurt & Koksal, 2025), the aim of the present study is to examine whether children’s emotional self-efficacy (trait-EI) could be related to eating behaviors through greater or lesser awareness of the eating moment and a better ability to use food for nutritional purposes and not as an emotional regulator.

In line with this, in the preliminary phase, the study aims to examine differences in the levels of the variables between boys and girls. Furthermore, any differences in levels of trait-EI and mindful eating between children who present high versus low levels of emotional over-eating are examined.

Finally, the specific objectives of the present study are to verify if: (a) children’s trait-EI is associated with their emotional over-eating; (b) children’s trait-EI is associated with their mindful eating; (c) mindful eating mediates the relation between trait-EI and emotional over-eating.

## 2. Method

### 2.1. Participants

This study involved 110 Italian families with children aged between 8 and 12 years (M = 9.68; SD = 1.29). All participants spoke Italian as their first language. In total, 51% of children were male, and 49% were female. The child group had a BMI range between 12.76 and 29.01 (M = 17.73; SD = 2.86), which falls within the normal weight category for children aged 8–12 years. Considering the male and female groups separately, the former has a mean BMI of 18.07 (SD = 3.21) while the latter has a mean BMI of 17.35 (SD = 2.41).

Regarding the group of fathers, ages ranged from 35 to 59 years (M = 45.69; SD = 5.66), and 90% declared that they are married and 5% that they cohabitate. The others declared themselves to be separate. Concerning education levels, 28% of them completed middle school, 58% had a high school diploma, 12% held a university degree; a small part of the sample had an elementary title (1%) or a postgraduate degree (1%). The fathers were asked to declare their employment, and the statistics show that 56% held dependent jobs, 36% worked independently, 5% were unemployed, and 1% of fathers were homemakers. The fathers’ BMIs ranged between 18.97 and 38.00 (M = 26.06; SD = 3.26), thus representing an overweight sample.

In the group of mothers, ages ranged from 25 to 54 years (M = 42.22; SD = 5.15). Regarding marital status, 90% of mothers answered that they were married, 5% that they cohabitated, and 5% that they were separated. Most of them held a high school diploma (56%), and the others had completed middle school (14%), a university degree (26%), or another qualification (4%). Regarding mothers’ employment, 32% held dependent jobs, 15% worked independently, 15% were unemployed, and 38% were homemakers. Considering that the mean of BMI for mothers was 24.14 (SD = 4.65), in general, they can be considered of normal weight.

No data were available on family income. However, based on the reported cities of residence, the majority of the sample appears to come from predominantly urban contexts (medium-sized provincial capitals located in Southern Italy).

### 2.2. Procedure

A convenience sampling technique was employed to solicit families to participate in this study. Specifically, the participation of pairs of parents and at least one child aged between 8 and 12 was required. Data were collected over the course of approximately two years. Before starting the study, authorization was obtained from the local ethics committee, and the ethical standards of the 1964 Helsinki Declaration and its later amendments were properly evaluated. In addition, before completing the research protocols, the participating parents received general information about the aims of the study and provided informed consent for themselves and their child. Parents could choose to complete the questionnaire either online, via a Google Forms survey, or in the traditional paper-and-pencil format. Given the children’s age, only the paper format was available for them. The researcher assigned the same ID code to the research protocol of each member belonging to the same family unit, which allowed participants’ data to be matched during the data analysis phase. The collected data were subsequently tabulated in an Excel spreadsheet and analyzed using the statistical software IBM-SPSS version 19 and RStudio version 4.1.1.

### 2.3. Measures

A personal data sheet revealed the demographic characteristics of the families. Furthermore, the frequency of consumption of main food categories (such as bread/pasta, milk/cheeses, meat/fish, etc.) was requested in order to detect the main eating habits of children and their parents.

The Trait emotional intelligence questionnaire—children short form (TEIQUE-CSF; Mavroveli et al., 2008a; Russo et al., 2012) was developed for the assessment of trait-EI in children through short sentences that concerned typical emotional reactions and management, and the ability to recognize the emotions of others. The scale consists of 36 items organized on a 5-point Likert scale from “1 = Completely Disagree” to “5 = Completely Agree”. The sentences are easily understood by children aged 8 and over. An example of an item might be “When I’m sad, I try to do something to change my mood.” The Italian validation of TEIQue-CF (Russo et al., 2012), which looked at the extended version of the questionnaire, demonstrated good structural (factorial) validity, with its nine facets loading on a general Trait-EI factor (explaining 39.1% of the variance), with factor loadings ranging from 0.39 to 0.76.

Convergent validity was supported by moderate correlations with the Big Five personality dimensions (r = 0.38–0.47), while discriminant validity was evidenced by the absence of a significant relationship with nonverbal intelligence (r = 0.06, ns) (Russo et al., 2012). Russo et al. (2012) also provided evidence of the criterion and incremental validity of the TEIQue-CF, showing that trait-EI predicted anxiety (β = −0.43) and depression (β = −0.59) and remained significant after controlling for the Big Five (βs = −0.23 to −0.34). In this study, the TEIQue-CSF was used, which is the short form version from Mavroveli et al. (2008a), which, although it has not been validated in the Italian context, has been used in recent studies conducted in Italy (Agnoli et al., 2023), showing a good internal consistency index for the total score of trait-EI (α = 0.81). Similarly, in this study, internal consistency analysis highlighted that the questionnaire displayed a good Cronbach’s alpha index, α = 0.87.

The Mindful Eating Questionnaire for Children (MEQ-C; Hart et al., 2018) was used to measure levels of mindful eating in children through the use of 12 items. The instrument is structured into two subscales, Awareness (4 items) and Mindless Eating (8 items); however, for the purposes of the present study, the total Mindful Eating scale was considered. The items are rated on a 4-point Likert scale, ranging from “1 = Never” to “4 = Always”. An example of an item is “I notice the flavors of my food.” Most items, which focus on food unawareness (e.g., “I eat too fast to taste my food”), were then recoded to obtain a positive overall Mindful Eating score. For the MEQ-C, the original article of validation by Hart et al. (2018) reported evidence of its content, structural, discriminant, convergent, criterion, and temporal validity. Content validity was supported by expert classification of items (79.7% agreement). Structural validity was demonstrated through exploratory factor analysis, identifying two factors (Awareness and Mindless Eating) with good model fit (CFI = 0.95, RMSEA = 0.03, SRMR = 0.04). Discriminant validity was supported by the very low correlation between factors (r = 0.02). Convergent validity was shown by significant correlations with emotional eating (r = 0.53), food craving (r = 0.42), and other unhealthy food consumption patterns (i.e., sugar-sweetened beverages, r = 0.16, and salty snacks, r = 0.25). Criterion validity was supported by regression models predicting eating behaviors (R^2^ up to 0.40). Test–retest reliability indicated temporal stability (Awareness r = 0.44; Mindless Eating r = 0.51). Reliability analyses in this study showed that the scale displayed a good Cronbach’s alpha index, α = 0.69. This value is slightly below the conventional 0.70 threshold, but remains within the range typically observed for brief self-report instruments in developmental samples (Peterson, 1994).

The Children’s Eating Behaviour Questionnaire (CEBQ; Wardle et al., 2001) is a questionnaire addressed to parents and used for the evaluation of eating habits in children and early precursors of obesity or eating disorders. The instrument has 35 items in total and is divided into the following subscales: Food responsiveness (5 items), Emotional over-eating (4 items), Enjoyment of food (4 items), Desire to drink (3 items), Satiety responsiveness (5 items), Slowness in eating (4 items), Emotional under-eating (4 items), Food fussiness (6 items). For the present study, attention was paid to only items related to the scale of Emotional Over-Eating. From the original validation study (Wardle et al., 2001), the CEBQ showed adequate evidence of content validity. The eight-factor structure was confirmed through principal component analysis (PCA), with unidimensional factors explaining 50–84% of the variance and eigenvalues > 1. The instrument also demonstrated good internal consistency (α = 0.74–0.91) and adequate test–retest reliability (r = 0.52–0.87). Parents rated the frequency of their child’s behaviors and experiences on a five-point Likert scale ranging from “1 = Never” to “5 = Always”. An example of an item is “My child eats more when worried”. The Cronbach’s alpha index for emotional over-eating in the group of fathers was α = 0.81, and in the group of mothers was α = 0.76

### 2.4. Data Analysis

**Figure 1:**
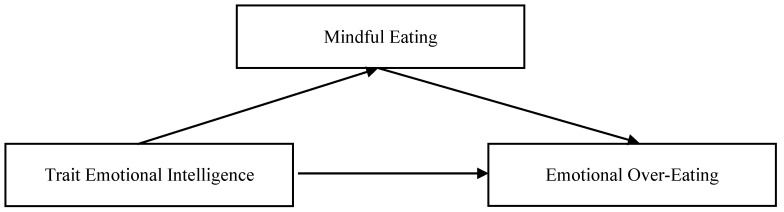
Hypothesized regression-based mediation model.

This study used the Statistical Package for Social Sciences (IBM SPSS Statistics 19) to conduct preliminary analyses (descriptive statistics, frequencies, reliability and correlations) and to test differences among groups. For this cross-sectional correlational study, in order to examine the hypothesized relations among the study variables, a regression-based mediation model was conducted through R software (version 4.1.1; R Core Team, 2013) run through R Studio (R Studio Team, 2017) with the lavaan package (Rosseel, 2012). See Figure 1.

## 3. Results

### 3.1. Descriptive and Correlational Analyses

First, preliminary analyses included descriptive statistics and frequencies for the main demographic characteristics of the participants, for the anthropometric measurements of family members (weight, height and BMI), and for the consumption of the main food categories. On the latter frequency analysis, from the reports of mothers, fathers and their children, it emerged that the participants presented a regular diet, in line with the Mediterranean diet model, demonstrating only an excessive consumption of sweets and snacks compared to the recommendations (WHO, 2025). See the Supplementary Materials for more details.

Furthermore, descriptive statistics (mean, standard deviations, skewness, kurtosis) for the examined variables were tested (Table 1). To evaluate the internal consistency of the measurement scales, reliability analyses were conducted using Cronbach’s alpha coefficient. The t-test for independent samples, in the preliminary phase, allowed us to identify that there were no significant differences between males and females in the levels of the variables (Trait-EI: t(108) = −0.07 *p* = 0.95; Mindful Eating: t(108) = −1.21 *p =* 0.23; Emotional Over-Eating: t(108) = 1.27 *p =* 0.21) or with respect to BMI, t(106) = 1.31 *p =* 0.19. Subsequently, using a t-test for independent samples, a difference was found in the levels of variables between the group of children who had high levels of emotional over-eating compared to those with low levels. In detail, to create the two groups, children with an average score below 2 were assigned to the “low emotional over-eating” group, whereas those with an average score above 2 were assigned to the “high emotional over-eating” group. Differences between these groups were then examined. Statistically significant differences emerged in trait-EI, t(108) = 2.56 *p =* 0.01 and Mindful Eating, t(108) = 5.40 *p* < 0.001. Specifically, the mean of trait-EI was higher in the group of children with low emotional over-eating (M = 3.75; SD = 0.49) than in those with high levels of emotional over-eating (M = 3.53; SD = 0.39). Even compared to Mindful Eating, children with low emotional over-eating had a higher mean (M = 3.24; SD = 0.38) than the group of children with high emotional over-eating (M = 2.82; SD = 0.43).

Pearson’s correlational analysis allowed to detect several significant associations between the study variables. In this step, children’s emotional over-eating was assessed separately from the mothers’ and fathers’ reports; however, since the two scores were significantly correlated (r = 0.72; *p* < 0.001) and represent indicators of the same construct, a composite index was used, given by the average score of the two measures for subsequent analyses.

Finally, before testing the hypothesized model to assess whether the mediation pattern was equivalent between males and females, an invariance analysis was conducted. The configural model, in which all paths were free to vary between groups, was compared with a structural model in which paths were constrained to be equal between the two groups. The scaled chi-square difference test (Satorra-Bentler) did not show a significant worsening of the constrained model compared to the configural one, Δχ^2^ (3) = 1.08, *p* = 0.78 (Satorra & Bentler, 2001). This indicates that the model is invariant between males and females, and that the estimated paths can be considered equivalent for both groups.

#### Regression-Based Mediation Model

**Figure 2:**
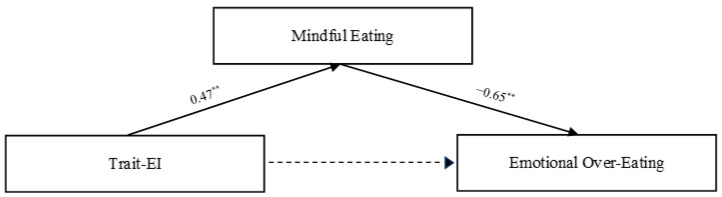
Significant association in the regression-based mediation model. Note. ** *p* < 0.01.

Secondly, to analyze the relationship between the variables under study, a regression-based mediation model was tested. The mediation model was tested using a path analysis with observed composite scores, and because the model is saturated (df = 0), global fit indices (e.g., CFI, RMSEA, SRMR) are not informative and therefore are not reported. Regression analyses showed that children’s trait-EI was positively associated with mindful eating (β = 0.47, *p* < 0.001). The latter, in turn, was negatively and statistically significantly associated with emotional over-eating (β = −0.65, *p* < 0.001). The direct effect of children’s trait-EI on emotional over-eating was not statistically significant (*p* = 0.09). See Figure 2 and Table 2.

## 4. Discussion

The general aim of the present study was to investigate the role of trait-EI in children’s emotional eating and its associations with mindful eating.

Our results only partially confirmed the first hypothesis. In fact, trait-EI was found to be related to children’s emotional over-eating, but this direct association was not statistically significant in the regression-based mediation model, where only an indirect association emerged, as discussed below. These results fit into a still-scant literature analyzing the association between trait-EI and eating behaviors among children. The lack of a direct association suggests the need to thoroughly investigate the mechanisms underlying the influence of trait-EI on eating outcomes, as these pathways may differ somewhat from those typical of adults (Biolcati et al., 2021; Cuesta-Zamora et al., 2018).

As regards the second objective, a positive and significant association was found between trait-EI and mindful eating in children. Children who generally have greater awareness of their emotions and are able to regulate them appropriately can extend these attitudes to the act of eating. This evidence supports previous studies showing that trait-EI and mindful eating are closely related in adults at the cross-cultural level (Hajar et al., 2020) and extends this evidence, for the first time, to a group of children in the Italian context.

The construct of mindful eating, where eating is driven by physical rather than emotional reasons, can in fact be considered conceptually opposite to emotional eating, where eating occurs in response to negative emotions and is characterized by a loss of control over the eating episode (Bennett & Latner, 2022). These considerations are also supported by the negative association that emerged, in the present study, between trait-EI and emotional over-eating in children, as reported by mothers and fathers. Emotional over-eating refers to excessive food intake aimed at alleviating unpleasant emotional experiences in children. In continuity with previous longitudinal studies showing that, in early childhood, the use of food as a tool for emotional regulation and as a reward by parents favors the development of emotional over-eating (Powell et al., 2017), current evidence suggests that, in the transition from an other-directed regulation mode to more self-regulated forms in emotional management, maladaptive strategies related to feeding may emerge in children with low trait-EI.

Another important finding concerns the negative association between mindful eating in children and emotional over-eating. Recently, a quasi-experimental study, in which a mindful eating intervention was conducted in pre-adolescents, demonstrated that, as eating awareness increased, emotional eating levels in children decreased (Kocaadam-Bozkurt & Koksal, 2025). The results of our study confirm previous data and contribute to the expansion of the literature on eating dynamics among children.

As regards the third objective, the main result of the present study concerns the identification of an indirect association between trait-EI and over-eating through the mediating role of mindful eating. Although trait-EI alone does not explain emotional over-eating in the regression-based mediation model, the relationship between the two variables is explained through mindful eating. In other words, children with a higher level of trait-EI are able to recognize, understand, and manage their emotions effectively and can relate to food with greater awareness and attention, paying attention to internal and external cues associated with the feeding moment. Children, therefore, eat by paying attention to signs of hunger and satiety, without automatically reacting to emotional states, and this mindful eating approach, in turn, may be negatively associated with resorting to food as a strategy to manage negative emotions through over-eating. This finding is innovative compared to the existing literature, since, although previous studies have already highlighted the relationship between emotional skills and mindful eating (Kidwell et al., 2015) and confirmed that successful emotion regulation protects against unhealthy emotional eating practices (Favieri et al., 2021; Giusti et al., 2021), to date, the role of trait-EI as a factor associated with children’s emotional over-eating through a mindful eating approach is still unexplored (Saptır & Turan, 2025). The rationale for these outcomes could be explained by the fact that a good level of trait-EI, which is characterized by good awareness of one’s emotional state, combined with the awareness strategies inherent to the mindful eating approach, could be linked to a reduced manifestation of emotional over-eating, thereby limiting uncontrolled eating behaviors; that is, all the automatisms that could lead to eating in response to emotional rather than physical sensations.

The latest differential results from this study also support the above evidence. Indeed, it was found that children with high levels of emotional over-eating had low trait-EI and low levels of mindful eating, while the group with low levels of emotional over-eating had greater trait-EI and greater awareness of the eating moment. With respect to gender differences in the levels of the variables, the lack of significant differences in trait-EI between boys and girls, in line with the studies by Mavroveli et al. (2008b), suggests that at these ages, emotional competencies may still be in a developmental phase and that, rather than gender, other temperamental characteristics may better explain interindividual differences (López-Cassà et al., 2022; Reyes-Wapano, 2021). Moreover, although recent studies have identified a tendency among boys toward more automatic eating patterns (Kocaadam-Bozkurt et al., 2022), these studies, in line with the results of the present study, do not report significant differences in levels of mindful eating. This finding is consistent with the results reported by Putri et al. (2024), who found only partial gender differences limited to specific dimensions of mindful eating, but no clear superiority of one gender over the other in overall mindful eating. Taken together, these findings may also be consistent with the absence of gender differences in emotional over-eating and BMI, in line with the broader evidence linking emotion regulation difficulties to unhealthy eating behaviors (Favieri et al., 2021).

### Limitations and Future Perspective

The study conducted explored recent constructs in a still under-investigated age group, taking into account data from the entire household (children and their parents). However, some methodological limitations should be taken into account when interpreting the results. First, with regard to the generalizability of the findings, the sample is limited to the Italian context only, and the sample size may need to be further expanded in future research. Furthermore, the lack of detailed socioeconomic information in this study, such as family income and parental education, should be considered. In fact, socioeconomic conditions are known to influence children’s eating behaviors through multiple pathways, including food access, parental resources, and health-related knowledge. Since some of the observed relationships may reflect unmeasured contextual differences, caution should be exercised when extending them to populations with different socioeconomic backgrounds.

Second, the cross-sectional nature of the study does not allow for an understanding of the direction of causality among the relationships; although trait-EI, due to its dispositional nature, could be considered a precursor of levels of mindful eating or emotional over-eating, this directionality should be confirmed in future longitudinal studies and with more sophisticated analytical models. Another aspect worthy of consideration concerns the fact that the present study relies on self-reported measures administered to participants aged between 8 and 12 years (in addition to parental measures). Although during pre-adolescence, children are generally able to provide information about their internal experiences, their ability to accurately recognize, understand, and verbalize complex emotional states and internal processes, such as awareness during mealtime, may be partial or unstable. Finally, the present study did not consider some potential factors that could influence the variables under study, such as parents’ trait-EI, their food-related educational practices, and their own levels of mindful eating.

## 5. Applicative Implications

This study presents significant applicative implications from a preventive and educational perspective. Trait-EI could represent a significant protective or vulnerability factor with regard to children’s eating behaviors and outcomes. Given the need to combat the increasingly widespread prevalence of childhood obesity and prevent the onset of eating disorders in childhood, it is important to implement interventions aimed at enhancing emotional skills and awareness in children from an early age. Trait-EI and mindful eating, in fact, represent key factors in the processes involved in regulating eating behaviors and, therefore, should be the focus of structured preventive interventions aimed not only at children but also at the family system as a whole in order to promote more adaptive ways of managing emotions and relationships with food.

## 6. Conclusions

In conclusion, the present study contributes to the existing body of literature by elucidating the probable protective role of trait emotional intelligence in relation to emotional eating, operating through increased levels of mindful eating. Although trait emotional intelligence does not show a direct association with emotional over-eating, it appears to be indirectly associated with it by promoting greater awareness and attentional regulation during children’s eating episodes. Moreover, these findings emphasize the importance of mindful eating as a central mechanism linking emotional competencies to eating behaviors in childhood.

## Figures and Tables

**Table 1:** Preliminary analyses.

	Min	Max	M	SD	Skew	Kurt.	a	2	3	4
1. Trait-EI	2.64	4.89	3.65	0.46	0.47	−0.33	0.87	0.47 **	−0.35 **	−0.28 **
2. Mindful Eating	1.92	4.00	3.06	0.45	−0.28	−0.33	0.69	-	−0.48 **	−0.35 **
3. Emotional Over-Eating (Fathers’ report)	1.00	4.00	2.10	0.89	0.39	−1.08	0.81	-	-	0.72 **
4. Emotional Over-Eating (Mothers’ report)	1.00	4.00	1.94	0.81	0.61	−0.48	0.76	-	-	-

Note. ** *p* < 0.01.

**Table 2:** Coefficients of the direct effects for the regression-based mediation model.

					95% CI for b		
Direct Associations	b	SE	z	*p*	LL	UP	β
Trait-EI → Mindful Eating	0.47	0.09	5.51	0.00	0.30	0.63	0.47
Mindful Eating → Emotional Over-eating	−0.65	0.16	−4.20	0.00	−0.96	−0.35	−0.37
Trait-EI → Emotional Over-eating	−0.28	0.17	−1.71	0.09	−0.61	0.04	−0.16

## Data Availability

The data presented in this study are available on request from the corresponding author due to privacy restrictions set forth by the institutional ethics board.

## Supplementary Materials

The following supporting information can be downloaded at: https://www.mdpi.com/article/10.3390/bs16020302/s1, Histogram S1: Frequencies of food consumption in children according to the paternal reports; Histogram S2: Frequencies of food consumption in children according to the maternal reports; Histogram S3: Frequencies of food consumption in children.
