# Supplementary material for: Trait Emotional Intelligence and Children’s Eating Practices
Source: Behav Sci (Basel). 2026 Feb 20;16(2):302. doi: 10.3390/bs16020302 (PMC12938787; doi:10.3390/bs16020302)
Supplement: Supplementary file 1 [file behavsci-16-00302-s001.zip › behavsci-4074392-supplementary.pdf]

Supplementary materials

Histograms 1- Frequencies of food consumption in children according to the paternal reports

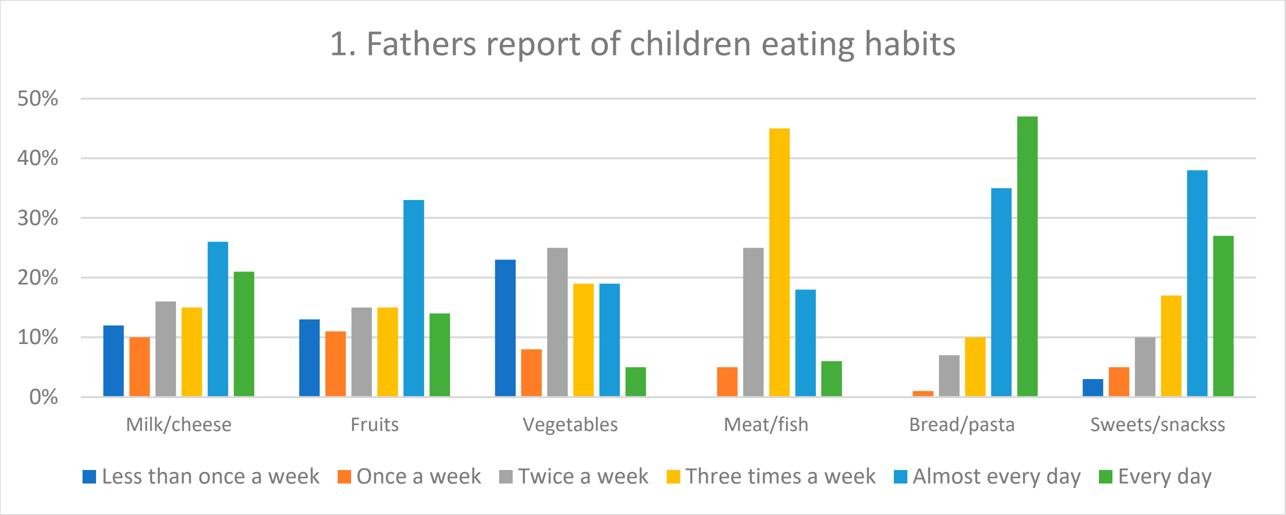

Histograms 2- Frequencies of food consumption in children according to the maternal reports

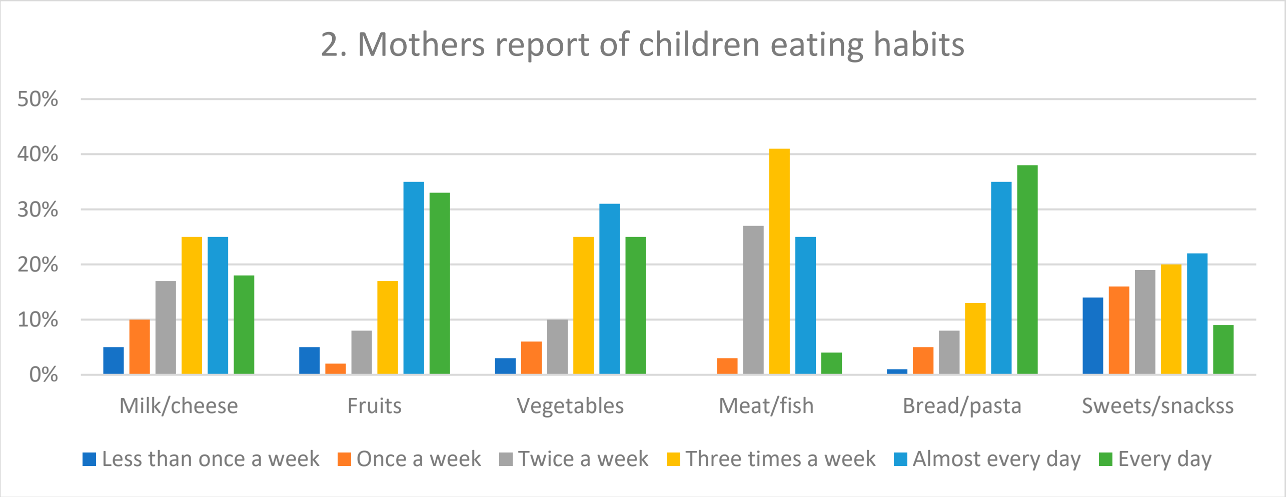

Histograms 3- Frequencies of food consumption in children

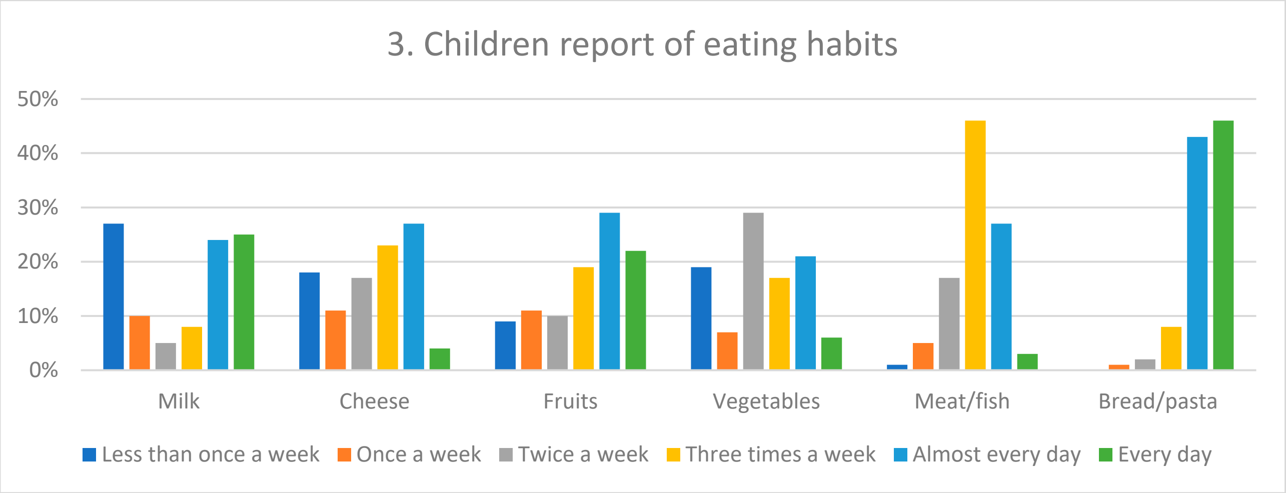

Brief description of the data that emerged. With respect to the frequencies of child food consumption, reports from mothers and fathers revealed a fairly regular pattern of food consumption in children, except for the consumption of sweets and snacks, which were consumed with high frequency. Fruit consumption occurs almost daily for most of the sample; the carbohydrate food category, represented by bread and pasta, is consumed daily by both maternal and paternal reports, and most children also consume meat and fish at least three times a week. Dairy products are consumed three to four times a week for most children, although with a more variable distribution between mother and father reports. Compared to vegetables, there is a slight disagreement between the reports of mothers and fathers: fathers consider the consumption of this food category as less frequent than mothers. Regarding the frequency of consumption of sweets and snacks, mothers report less frequent consumption than fathers, but in both reports, most children consume high-calorie foods almost every day.

Considering the children's reports, milk consumption occurs with a very heterogeneous frequency, a large part of the sample claims to drink it every day while another large part claims to never drink it. A similar situation occurs for dairy consumption. Vegetables, according to children, are consumed less frequently than parents report. Compared to fruit, meat/fish, and bread/pasta consumption, children's data mirror parents' reports; the majority of the sample reported eating fruit "almost every day," meat and fish "three times a week," and bread and pasta "almost every day" to "every day". No information on sweet/snack consumption was obtained directly from children.
